# A neuromorphic dataset for tabletop object segmentation in indoor cluttered environment

**DOI:** 10.1038/s41597-024-02920-1

**Published:** 2024-01-25

**Authors:** Xiaoqian Huang, Sanket Kachole, Abdulla Ayyad, Fariborz Baghaei Naeini, Dimitrios Makris, Yahya Zweiri

**Affiliations:** 1 Advanced Research and Innovation Center (ARIC), Khalifa University, Abu Dhabi, UAE; 2 Khalifa University Center for Autonomous Robotic Systems (KUCARS), Khalifa University, Abu Dhabi, UAE; 3 School of Computer Science and Mathematics, Kingston University, London, UK; 4 Department of Aerospace Engineering, Khalifa University, Abu Dhabi, UAE

**Keywords:** Engineering, Optics and photonics, Aerospace engineering

## Abstract

Event-based cameras are commonly leveraged to mitigate issues such as motion blur, low dynamic range, and limited time sampling, which plague conventional cameras. However, a lack of dedicated event-based datasets for benchmarking segmentation algorithms, especially those offering critical depth information for occluded scenes, has been observed. In response, this paper introduces a novel Event-based Segmentation Dataset (ESD), a high-quality event 3D spatial-temporal dataset designed for indoor object segmentation within cluttered environments. ESD encompasses 145 sequences featuring 14,166 manually annotated RGB frames, along with a substantial event count of 21.88 million and 20.80 million events from two stereo-configured event-based cameras. Notably, this densely annotated 3D spatial-temporal event-based segmentation benchmark for tabletop objects represents a pioneering initiative, providing event-wise depth, and annotated instance labels, in addition to corresponding RGBD frames. By releasing ESD, our aim is to offer the research community a challenging segmentation benchmark of exceptional quality.

## Background & Summary

In the 4^th^ Industrial Revolution, there is a substantial surge in the demand for multifunctional robots. Gripper-equipped robots have gained popularity and are pivotal for grasping tasks in various industries. They offer the manufacturing industry a distinct advantage by reducing production time and enhancing overall throughput. A major portion of these tasks necessitates robots to exhibit competence in handling objects of diverse shapes, weights, and textures. However, it is worth noting that most techniques are utilized to train robots for tasks suited to structured environments, where prior knowledge of the scene and objects is readily available. Such tasks are prone to significant errors and present substantial challenges in achieving full automation, particularly within unstructured environments^1^. In unstructured environments, objects are disordered, and exhibit unknown shapes and geometries, thereby requiring robotic systems to rely on real-time perception and comprehension through robotic vision. This is in stark contrast to the structured environment where prior knowledge and object models can be employed. Consequently, the key to addressing this challenge lies in robotic perception, which enables robots to localize, segment, and grasp objects in unstructured settings.

At present, most vision-based applications and research predominantly rely on traditional vision sensors such as RGB and RGBD sensors. Nonetheless, conventional frame-based cameras exhibit notable limitations, including high power consumption and extensive storage demands stemming from continuous full-frame sensing and data storage.Furthermore, their characteristics of low sampling rates and susceptibility to motion blur can adversely impact the perceptual quality for many vision-based applications. For instance, the conventional RGB camera’s low sampling rate results in motion blur when capturing images of fast-moving objects on conveyor belts within production lines^2^. Thus, the accuracy and success rate of object picking and placing are reduced at the perceiving stage. Neuromorphic vision sensors draw inspiration from biological systems, particularly the visual capabilities of fly eyes, which can parallelly and in real-time sense data with a microsecond-level sampling rate^3,4^. Leveraging these unique properties of event cameras, an increasing amount of research is exploring the applications of neuromorphic vision technology to mitigate motion blur and enhance efficiency in various domains. These applications encompass object tracking^5^, depth estimation^6^, autonomous driving^7^, and robotic grasping^8–11^.

In perception-related tasks, segmentation serves as a foundational pre-processing step, crucial for estimating the attributes of individual objects. This is particularly vital in vision-based robotic grasping applications, where the accurate localization and geometric details of each object are essential for formulating precise grasping strategies^12^. In other words, the quality of perception and segmentation has a direct and substantial impact on the quality of the grasping process. In recent years, learning-based approaches to segmentation and other vision-based tasks triggered a massive surge. Datasets are significant for computer vision supervised learning methods^13^. Moreover, datasets allow the comparison among various algorithms to provide benchmarks^14^. Several RGB and RGBD-based segmentation datasets were constructed to provide ground truth for the training and evaluation of deep-learning-based segmentation approaches. For instance, EasyLabel^15^ offers instance segmentation RGB-D dataset with point-wise labeled point-clouds information for cluttered objects in an indoor environment, where the depth height and the objects in clutter are varied. Also, synthetic dataset TOD was generated for unknown object segmentation^16^. Besides, there are many other public conventional datasets, such as MSCOCO^17^, PascalVoc^13^, and CityScape^18^ for multiple tasks including segmentation, object detection, and classification. In addition, amounts of conventional vision-based objects segmentation approaches were developed, such as FCN^19^, U-NET^20^, and DeepLab^21^ are commonly utilized as evaluation benchmarks.

However, research on event-based segmentation is still in the primary stage of development. In contrast to the booming research in instance segmentation using conventional frame-based vision, limited attention has been devoted to event-based instance segmentation of tabletop objects. Current solutions for event-based instance segmentation are commonly based on clustering. For example, certain event-based mean shift clustering methods were introduced in prior works^11,22^ that utilize 2D spatial and temporal information. Nonetheless, these methods encounter difficulties in segmenting occluded objects. To circumvent this limitation, the inclusion of depth information from RGBD imagery can prove advantageous. Furthermore, events complemented by depth information can serve as the ground truth for deep learning-based depth estimation approaches, such as spiking neural networks-based depth estimation from mono event camera^23^. However, there is a conspicuous absence of deep learning approaches for neuromorphic segmentation of tabletop objects. This shortage can be attributed to the insufficient availability of labeled data required for both training and testing. Instead of developing of novel instance segmentation methodologies, transfer learning from semantic segmentation networks could be a feasible and expedited approach to accomplish instance segmentation tasks. There are several approaches targeting event-based semantic segmentation for autonomous driving, such as EV-SegNet (2019)^24^, VID2E (2019)^25^, EVDistill (2021)^26^, EV transfer (2022)^27^, and ESS (2022)^28^. However, features provided by pure events are limited compared to RGB frames. The cross-modal networks, such as SA-GATE^24^ and CMX^29^, are being investigated nowadays to obtain abundant information from both events stream and complementary RGB frames.

To address this gap, we constructed an Event-based Segmentation Dataset (ESD) of tabletop objects in cluttered scenes, the first of its kind, providing event-wise depth and label information. Each sequence of ESD represents different challenges arising from various light conditions, occlusion conditions of objects, moving speeds, and moving trajectories of cameras. Furthermore, our dataset contains both RGBD frames and events data. Recent research has demonstrated that RGB frames can enhance the quality of events by augmenting the available pixel features^24,29,30^. The fusion of events and frames offers greater flexibility and increased potential for hybrid algorithm development, as opposed to relying solely on either pure event-based or frame-based techniques by using our dataset. Moreover, our dataset holds the distinction of being the first event-wise labeled neuromorphic dataset designed specifically for tabletop object segmentation. Tabletop datasets are essential for industrial sorting and grasping tasks. They offer a diverse range of objects and scenarios, allowing robots to acquire skills for a wide range of industrial challenges. Moreover, these datasets promote effective robot generalization and serve as valuable benchmarks for performance evaluation. Even if there are several recently released event-based segmentation, such as DDD17^31^ and DSEC-Semantic^28^ proposed for outdoor driving semantic segmentation, and EVIMO2^32^ and M3ED^33^ designed for motion segmentation instead of tabletop object segmentation.

Specifically, our dataset comprises two distinct subsets, namely ESD-1 and ESD-2, which are designated for training and testing purposes, particularly aimed at addressing unseen object segmentation tasks. These subsets encompass event streams, RGB frames, and depth information, all acquired using two Davis 346c event cameras and an Intel D435 RGBD camera affixed to the end effector of a UR10 robot. The ESD dataset contains events streams and grayscale frames (346 × 260) from event cameras, raw RGB frames (1080 × 720) and depth maps from RGBD cameras, moving speed and position of the end effector of UR10 robot. Data were collected under various conditions including different objects, camera movement speeds and trajectories, lighting conditions, and varying distances between the cameras and the tabletop. Events are labeled with depth information, and RGBD frames from the conventional camera are also provided in our dataset. Moreover, we rigorously evaluate several widely used segmentation methods on our proposed ESD to demonstrate the challenges.

This paper targets constructing a neuromorphic dataset for object segmentation since no previous datasets of this kind can be found in the literature. The rest of this paper is organized as follows. Section *Methods* explains the methods utilized to construct this dataset, including the designed experimental setup and protocol for collecting the dataset are presented in subsection *Experimental setup* and *experimental protocol*, respectively. Subsection *Image and events annotation* elaborates on the annotation methods for both RGB images and raw events data. Section *Data Records* describes the dataset format and attributes. Additionally, the constructed dataset was technically validated, and benchmarking of segmentation by the state-of-the-art approaches is provided in Section *Technical Validation*.

## Methods

### Experimental setup

As mentioned in Section *Background & Summary*, the availability of depth information is crucial in the context of the segmentation task, especially when addressing objects affected by occlusion. Depth information in the vision system can be acquired through various methods. The most common approach involves the use of a stereo camera setup, which calculates depth through triangulation by comparing disparities between corresponding points in the images^34,35^. Additionally, Multi-View Stereo (MVS) techniques leverage multiple images of a scene from different viewpoints, even with a single camera^36,37^. With the development of machine learning and deep learning, monocular depth estimation has recently gained high attention. These models are trained on extensive datasets to predict depth from single images, leveraging features, context, and object relationships^38,39^. Some cameras are equipped with integrated depth sensors, which may be based on stereo vision, Time-of-Flight (ToF) technology, or other methods to provide depth information alongside traditional RGB imagery. Therefore, we proposed this dataset to enable the community to test different methods of depth estimation for the segmentation task, and that’s why we provide both stereo event camera configuration and RGBD.

Specifically, mono event-based camera does not directly get this depth information, so in our dataset, the depth associated with each event is derived through a transformation process that maps the depth information from the RGBD camera frame into the event coordinate system. Therefore, by providing event-wise depth information, our dataset can facilitate the development of algorithms for segmentation that utilize depth information with mono event-based cameras. Moreover, another significant advantage resides in its applicability to real-time depth estimation tasks. Through the use of stereo event cameras, depth information can be derived through coordinated mapping. Furthermore, the inherently dynamic nature of event-based cameras opens the possibility of depth calculation even with mono event-based camera. The depth map captured from RGBD camera in our dataset can serve as the ground truth. Thus, by offering a dataset sourced from a stereo event camera system and depth information from RGBD camera, our dataset can be employed in a wide array of applications beyond segmentation tasks, such as depth estimation, optical flow and 3D reconstruction.

**Fig. 1:**
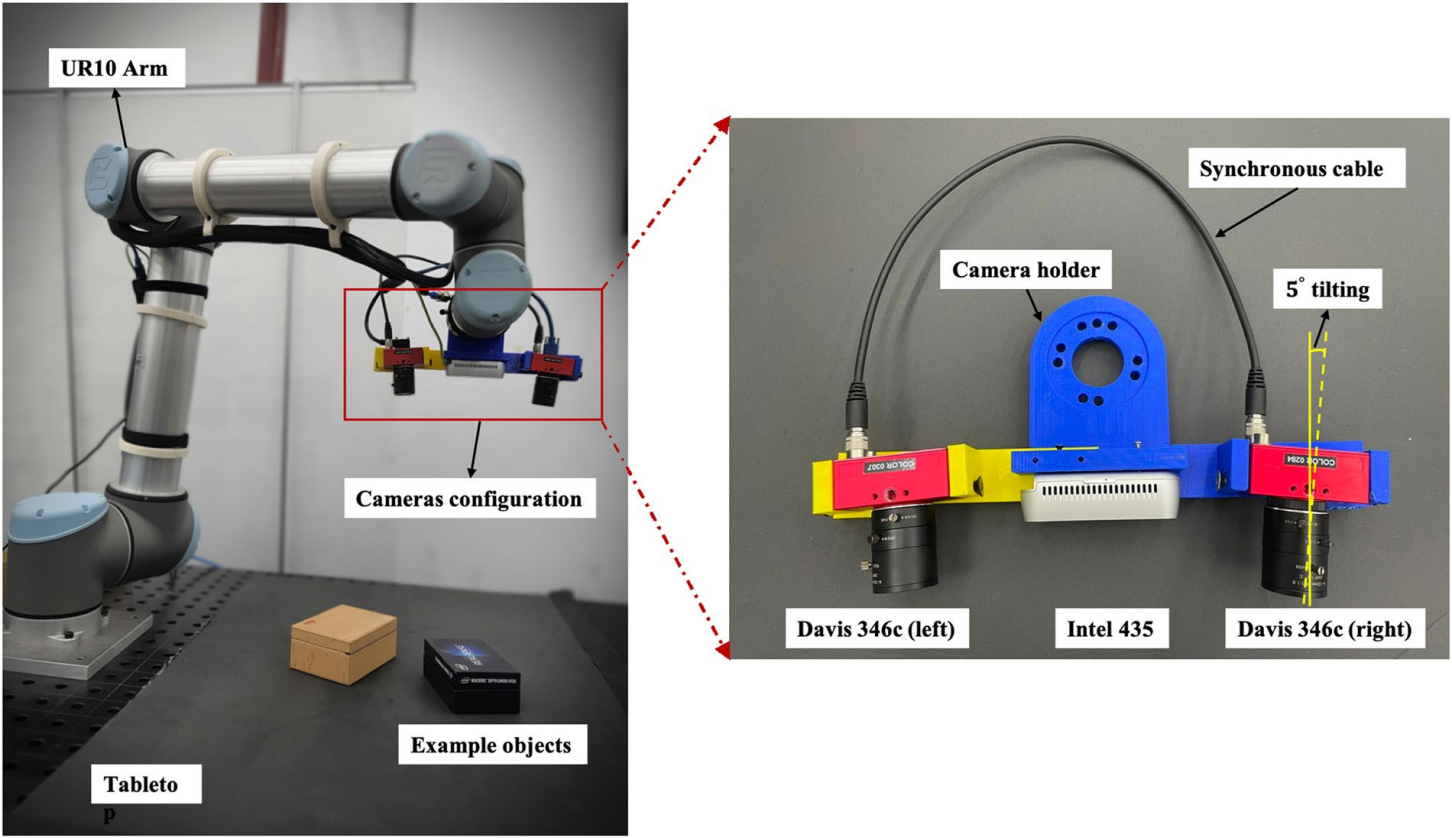
Hardware setup. Experimental hardware setup (left-side figure): three cameras are fixed on the end-effector of the UR10’s manipulator. Camera configuration (right-side figure): The RGBD camera *Intel D435* is placed in the middle, and two event-based cameras *Davis 346c* are mounted on the left and right sides with a tiled angle of 5 degrees towards the middle.

The hardware setup is built on the UR10 robot, as it can provide flexible and stable control of the camera’s movement with positional repeatability of 0.1 *mm*. Three cameras, including one RGBD camera *Intel D435* and two event cameras *Davis 346c*, are fixed in the camera holder attached to the robot’s end-effector. The overview of the setup is illustrated in the left image of Fig. 1.

To ensure the complete overlap of left and right cameras, the relative tilt angle between the two event cameras is calculated as 5 degrees with the assumed height of 0.82 *m*. Therefore, the two event cameras are tiled with 5 degrees towards the RGBD camera. Furthermore, synchronization connectors are employed to synchronize the two event-based cameras, ensuring that events are triggered simultaneously. Given the microsecond-level sample rate, synchronization between the event cameras and the RGBD camera is achieved by identifying the nearest timestamps.

### Experimental protocol

We collected and assembled the Event-based Segmentation Dataset (ESD) into two distinct subsets: ESD-1, designated for training purposes, and ESD-2, reserved for testing, primarily focusing on unseen object segmentation tasks. The training dataset encompasses up to 10 objects, whereas the testing dataset includes up to 5 objects. The data sequences were collected under various experimental conditions, which will be elaborated on in the subsection *Dataset Challenging Factors and Attributes*. These conditions encompass different object quantities (ranging from 2 to 10 objects in ESD-1 and 2 to 5 objects in ESD-2), varying lighting conditions (normal and low light), heights between cameras and tabletop (0.62 meters and 0.82 meters), occlusion conditions (with and without occlusion), varying camera movement speeds (0.15 m/s, 0.3 m/s, and 1 m/s in ESD-1, and 0.15 m/s and 1 m/s in ESD-2), as well as different camera trajectories (linear, and rotary and general motion in ESD-1, where general motion is a combination of linear and rotary motion, and linear and rotary motion in ESD-2). Furthermore, it is noteworthy that the objects present in ESD-1 differ from those in ESD-2, thereby rendering this dataset can be used for addressing challenges related to unknown object segmentation.

**Fig. 2:**
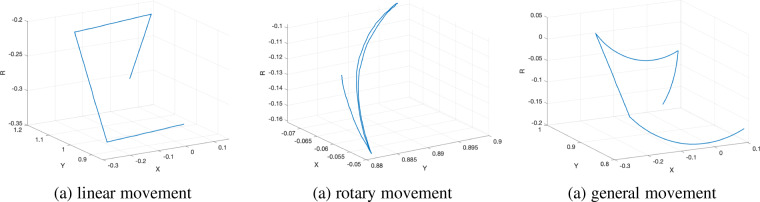
Designed moving trajectories in *x*–*y*–*r* space, where *x*–*y* indicates the plane that cameras move on, and the rotation is denoted in *r* axis.

Before conducting experiments to collect data, all of the event cameras and the RGBD camera were calibrated to obtain the intrinsic and extrinsic parameters^40^, crucial for subsequent data processing and annotation. Then setting up the specific conditions for each particular experiment, such as the height of cameras and the lighting condition. The UR10 robot’s end-effector, carrying the cameras, consistently started from the same position for all experiments. Generally, the majority of experimental setups for robotic grasping adopt an “eye-in-hand” configuration^10,41^, where the camera is attached to the end effector. In scenarios involving object pick-and-place tasks, the camera commonly maintains a downward orientation focusing on the tabletop. Consequently, manipulating the end effector at various speeds and trajectories, along with the attached camera, facilitates the simulation and replication of conditions encountered in robotic manipulation. Additionally, in event-based camera observation, an issue arises when no events are detected as the camera’s motion direction aligns parallel to object edges. Therefore, we formulated three distinct motion patterns: linear motion, most affected; general motion, less affected due to rotation; and pure rotary motion, effectively eliminating this issue. Furthermore, in many table-top grasping scenarios, event camera has an “act-to-perceive” nature, where the resulting events depend heavily on the linear and angular velocity of the camera, and not just the scene. That is why it is important to generate events using different camera motion types to generate a sufficient dataset that can be used to train models capable of generalization. Figure 2 illustrates the three designed different moving trajectories in *x*–*y*–*r* space using quaternion, where *x*–*y* indicates the plane that the cameras move on, and the rotation is denoted in *r* axis.

Overall 115 experiments and 30 experiments were conducted for ESD-1 and ESD-2, respectively. The RGB part of the dataset consists of 14,166 annotated images. In total, 21.88 million and 20.80 million events from the left and right event-based cameras were collected, respectively.

**Fig. 3:**
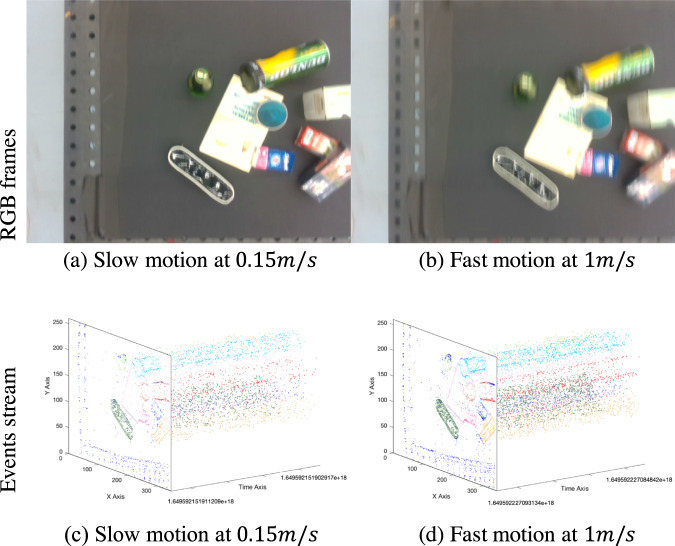
Visualization of RGB frames captured from RGBD camera and events streams obtained by event-based cameras in linear motion in the same time interval.

To measure the differences between similar frames, the difference between two adjacent frames is calculated by Root Mean Square Error (RMSE) of pixel values, which is a measure of the average pixel-wise difference providing a single scalar value representing the overall dissimilarity between the frames. The RMSE for the entire ESD dataset is 62.31. Additionally, we calculate RMSE for two specific scenarios: one involving a sequence with the slowest movement, consisting of two objects with fewer features, and the other with the fastest movement, involving ten objects with more features. The calculated RMSE values for these scenarios are 55.17 and 65.61, respectively. We also quantitatively evaluated the difference of consecutive RGB frames of widely used and newly released video datasets DAVIS^42^, MOSE^43^ and CLVOS23^44^. The calculated average RMSEs are 30.20, 28.69 and 26.27, respectively. By comparing the difference RMSE between our dataset and recently published datasets, we demonstrate that our dataset has sufficient variation in the collected images. This variation would supplement the generalization capabilities of machine learning models trained on our dataset.

1 $$RMSE=\sqrt{\frac{1}{n-1}{\sum }_{i=1}^{n-1}\frac{{\left({I}_{i+1}-{I}_{i}\right)}^{2}}{N}}$$

where *I*_*i*_ and *I*_*i* + 1_ denote the *i th* and (*i* + 1) *th* images. The total number of images is described as *N*. Additionally, even if consecutive frames look similar, the same does not necessarily apply to events. For example, performing the same linear motion at different speeds could result in different RGB frames, but the associated events data would exhibit significant differences. As depicted in Fig. 3a,b show the RGB visualization captured from the RGBD camera which looks quite similar to each other. However, when we examine the corresponding events stream within 1 ms in x-y-t coordinate, a substantial difference becomes evident as shown in (c,d). The discrepancy arises because events are generated asynchronously, leading to variations in temporal information captured by the events data.

### Image and events annotation

We tested different methods for the automatic annotation of RGB images and event data. Due to different features appearing with different perception angles of the camera, achieving precise automatic labeling of RGB images is quite challenging. Consequently, we manually labeled all RGB frames and utilize these manual annotations as references for the automatic annotation of event-based data.

#### Manual annotation of RGB frames

Our proposed ESD dataset contains 11,196 images for training and 2,970 images for testing in total. We used the online web annotator CVAT^45^ to manually annotate the tabletop objects in each frame. CVAT offers automatic features for pixel labelling. The polylines tool is used to draw the boundaries around the objects. Dealing with occlusion is one of the challenges of annotating this dataset. The occluded object is declared as the background whereas the front object is declared as the foreground.

**Fig. 4:**
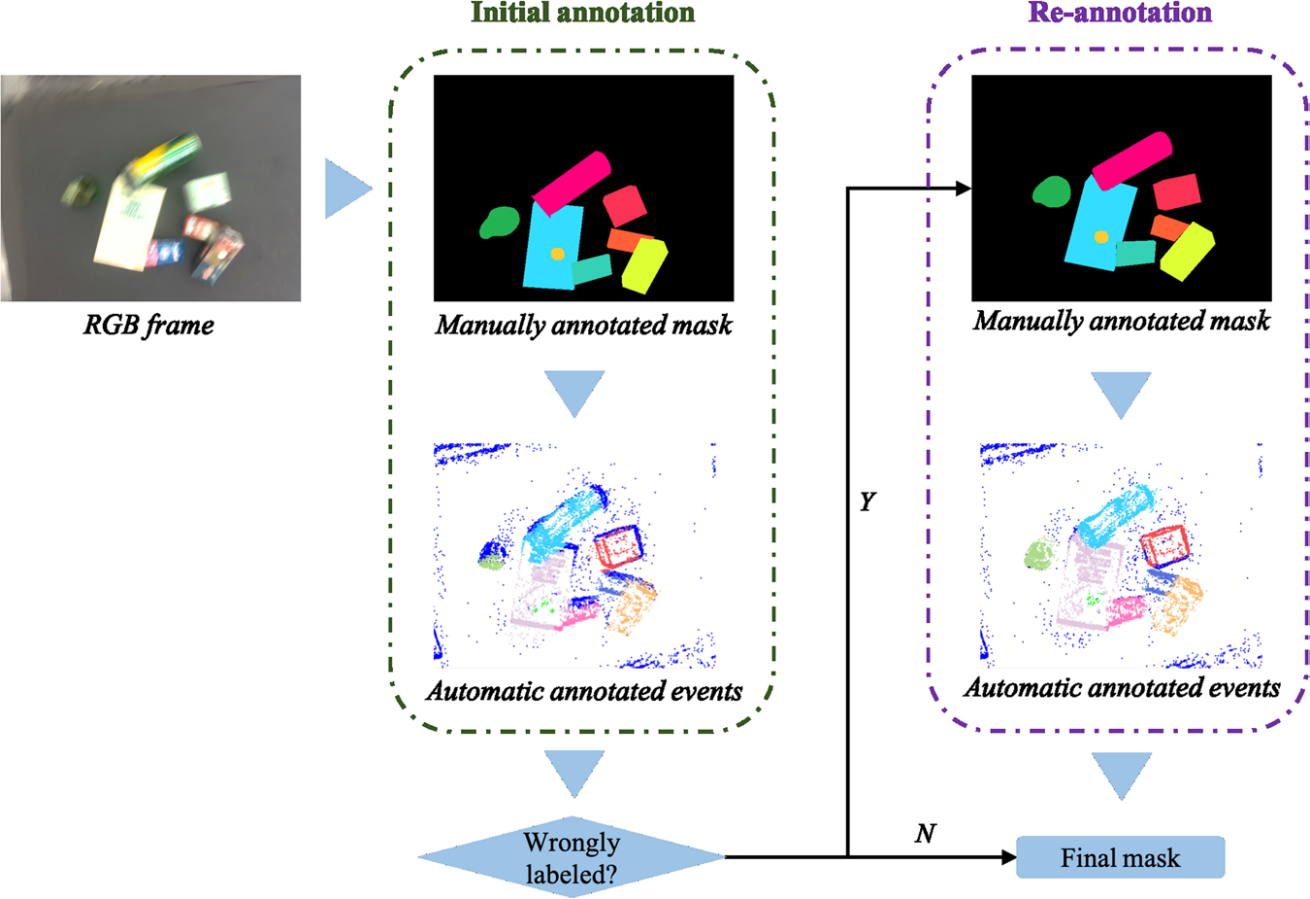
Two steps of labeling blurred images: initial annotation and re-annotation. If wrong labels show in the event frame, the second-round labeling of the RGB mask will be triggered according to the initial annotated events.

Furthermore, the motion blur resulting from the low sampling rate of the RGBD camera introduces ambiguity when manually labeling the boundaries of objects. Therefore, we addressed this challenge by conducting a two-step labeling process, as illustrated in Fig. 4. The initial annotation was established based on manual inferences of the objects’ positions in accordance with the trajectory of camera movement. Following this, once the corresponding events were fitted and annotated (as elaborated in section *Automatic labeling of events data*), we can observe the events frame to understand whether the mask frame had been accurately labeled with well-defined shapes and object outlines. If the event frame exhibited precise annotation, the initial annotated mask was retained as the final version. However, if the event frame indicated imprecision, we initiated the second step, involving the re-labeling of the RGB frame, continuing this process until the events were meticulously annotated.

#### Automatic labeling of events data

Events are labeled according to the annotated RGB masks, and the Pseudo code of automatic annotation of a sequence of events captured in one experiment as described in Algorithm 1.

##### Algorithm 1

Automatic Annotation for Events Data.

Events recorded can be considered as a continuous data stream with a high frequency (few microseconds). Thus, we divided sequences of events into intervals “*E*” of around 60 *ms* which is the same sampling period of the RGBD camera by finding the nearest timestamp between events and the RGB frame. Simultaneously, annotated mask frames “*S*” in RGBD coordinate are transformed to events coordinates as “*S*_*e*_” as described in Eqs. 2–4. First, the forward projection is applied to transform mask frames “*S*” in RGBD coordinate into RGBD camera coordinate as “*S*_*c*_” using the camera intrinsic parameters (Eq. 2). As expressed in Eq. 3, the coordinate transformation is applied twice to transform “*S*_*c*_” into world coordinate and event camera coordinate in sequence using the cameras’ extrinsic parameters. Building on that, masks in event camera coordinate are backward projected into events coordinate as described in Eq. 4.

2 $$\left\{\begin{array}{c}x=(u-{c}_{x})z/{f}_{x}\\ y=(v-{c}_{y})z/{f}_{y}\end{array}\right.$$

3 $$\left[\begin{array}{c}{x}_{e}\\ {y}_{e}\\ {z}_{e}\\ 1\end{array}\right]={\left[\begin{array}{cc}{{\bf{R}}}_{{\bf{e}}} & {{\bf{T}}}_{{\bf{e}}}\\ 0 & 1\end{array}\right]}^{-1}\left[\begin{array}{cc}{\bf{R}} & {\bf{T}}\\ 0 & 1\end{array}\right]\left[\begin{array}{c}x\\ y\\ z\\ 1\end{array}\right]$$

4 $$\left\{\begin{array}{c}{u}_{e}={f}_{xe}{x}_{e}/{z}_{e}+{c}_{xe}\\ {v}_{e}={f}_{ye}{y}_{e}/{z}_{e}+{c}_{ye}\end{array}\right.$$

where (*x*, *y*, *z*), (*u*, *v*), (*x*_*e*_, *y*_*e*_, *z*_*e*_), (*u*_*e*_, *v*_*e*_) and (*X*, *Y*, *Z*) represent the same point in RGBD camera coordinate, RGB image plane, event camera coordinate, events image plane, and world coordinate systems respectively. ${c}_{x},{c}_{y}$ and ${c}_{xe},{c}_{ye}$, indicate the center points in RGB and events image planes, respectively. Similarly, ${f}_{x},{f}_{y}$ and ${f}_{xe},{f}_{ye}$ denote the focal length of RGBD and event camera, respectively. **R** and **T** express the rotation matrix and translation vector from the RGBD camera coordinate to the world coordinate system. **R**_**e**_, **T**_**e**_ describes the rotation matrix and translation vector from the event camera coordinate to the world coordinate system.

However, the events recorded asynchronously appear in different locations since the camera keeps moving. Thus, events between two consecutive RGB frames are sliced into sub-intervals with 300 events “*E*_*ij*_”. Then fitting transformed event mask “*S*_*e*_” into events coordinate as “*S*_*et*_” by applying the Iterative Closest Point (ICP) algorithm^46^ to find the rigid body transformation between the two corresponding point sets *X* = {*x*_1_, *x*_2_, ‥, *x*_*n*_} and *P* = {*p*_1_, *p*_2_, ‥, *p*_*n*_}. The ICP algorithm assumes that the corresponding points *x*_*i*_ and *p*_*i*_ are the nearest ones, so the working principle is to find the rotation matrix *R* and translation *t* that minimizes the sum of the squared error *E*(**R,** **T**) as expressed in Eq. 5.

5 $$E(R,T)=\frac{1}{{N}_{p}}\mathop{\sum }\limits_{i=1}^{{N}_{p}}{\left\Vert {x}_{i}-{R}_{{p}_{i}}-t\right\Vert }^{2}$$

**Fig. 5:**
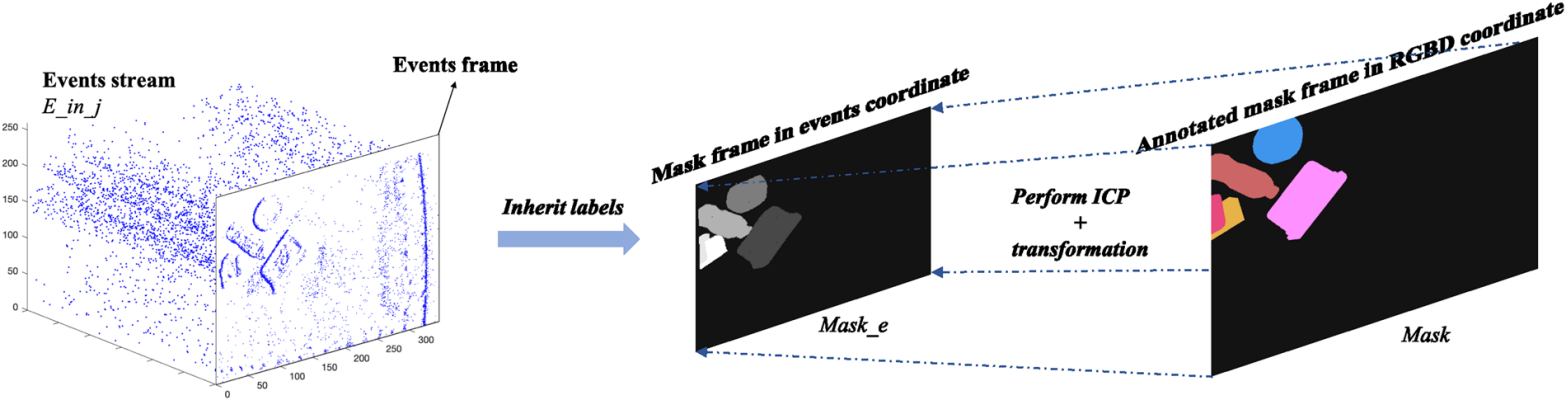
Principle of mapping the interval of events on the RGB frame coordinate for annotation.

Leveraging the transformation matrix derived from rotation and translation, calculated through ICP (Iterative Closest Point), the corresponding location of events on annotated mask frames will be obtained as ${x}_{i}^{{\prime} }=[{\bf{RT}}]{x}_{i}$. Therefore, the labels assigned to pixels on the RGB mask are inherited and applied to the events as well. The working principle is illustrated in Fig. 5.

In our setup, we position two event cameras and an RGBD camera in different locations, as illustrated in Fig. 1. Consequently, their views may not entirely overlap due to constraints on the distance between objects and the cameras. As a result, areas exclusively sensed by the event camera may be erroneously labeled as background, even when they correspond to actual objects. To tackle this issue, we crop out the blind area which is only sensed by the event camera. The event-based camera not only captures spatial information but also temporal information. As a result, the event occurrences that are cropped at a specific timestamp persist in subsequent timestamps, making them amenable to further processing.

### Data visualization

**Fig. 6:**
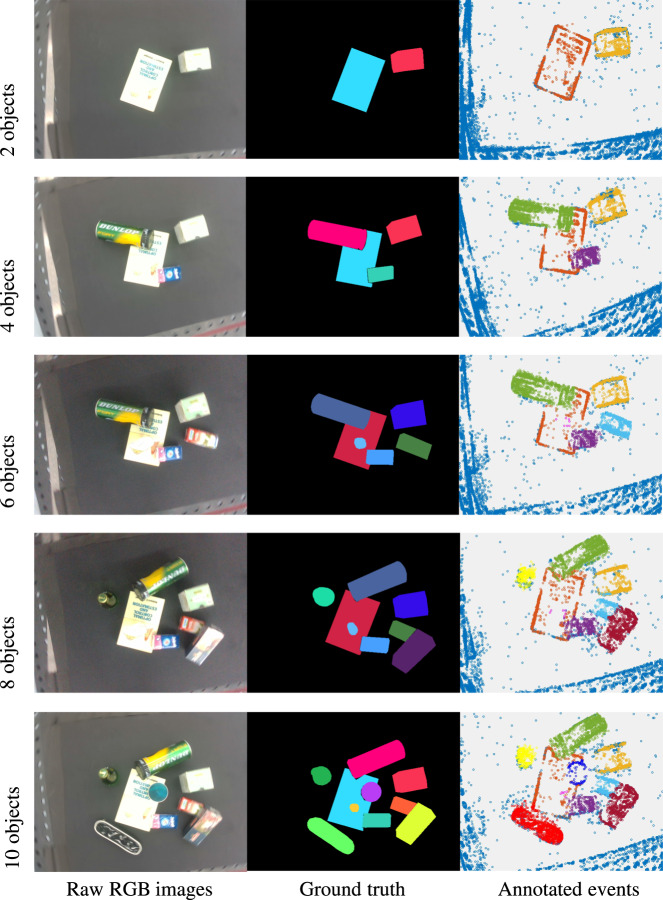
Example of the ESD-1 in terms of the number of objects attributes, under the condition of 0.15 moving speed, normal light condition, linear movement, and 0.82 height. Different colors in the RGB ground truth and annotated event mask mean different labels. Better view in color.

**Fig. 7:**
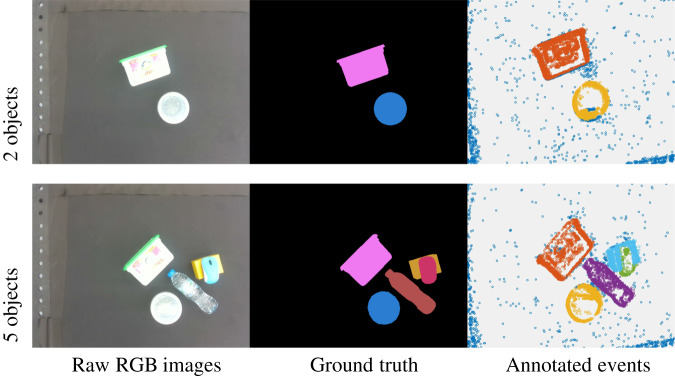
Example of unknown objects ESD-2 dataset in terms of the number of objects attributes, under the condition of 0.15 moving speed, normal light condition, linear movement, and 0.82 height. Different colors in the RGB ground truth and annotated event mask mean different labels. Better viewed in color.

We partitioned ESD into training (ESD-1) and testing (ESD-2) subsets for unseen object segmentation tasks. Training and testing dataset consists of up to 10 objects and 5 objects respectively. Notably, the testing dataset presents a challenge in addressing unseen object segmentation tasks, as it features different objects compared to the training dataset, ESD-1. Data sequences were collected under various experimental conditions which will be discussed in detail in subsequent subsection *Dataset challenging factors and attributes*. Examples of ESD-1 in terms of the number of objects’ attributes can be visualized in Fig. 6. The raw RGB image, annotated mask, and the corresponding annotated events (*N* = 3000) are illustrated for conditions of the different number of objects. Particularly, in the clusters of 2 objects, both the objects (ie. Book and box) are distanced from each other. For clusters of more than 2 objects, there are occlusions among objects. Besides, examples of ESD-2 for unseen object segmentation in terms of other attributes are depicted in Fig. 7.

## Data Records

**Fig. 8:**
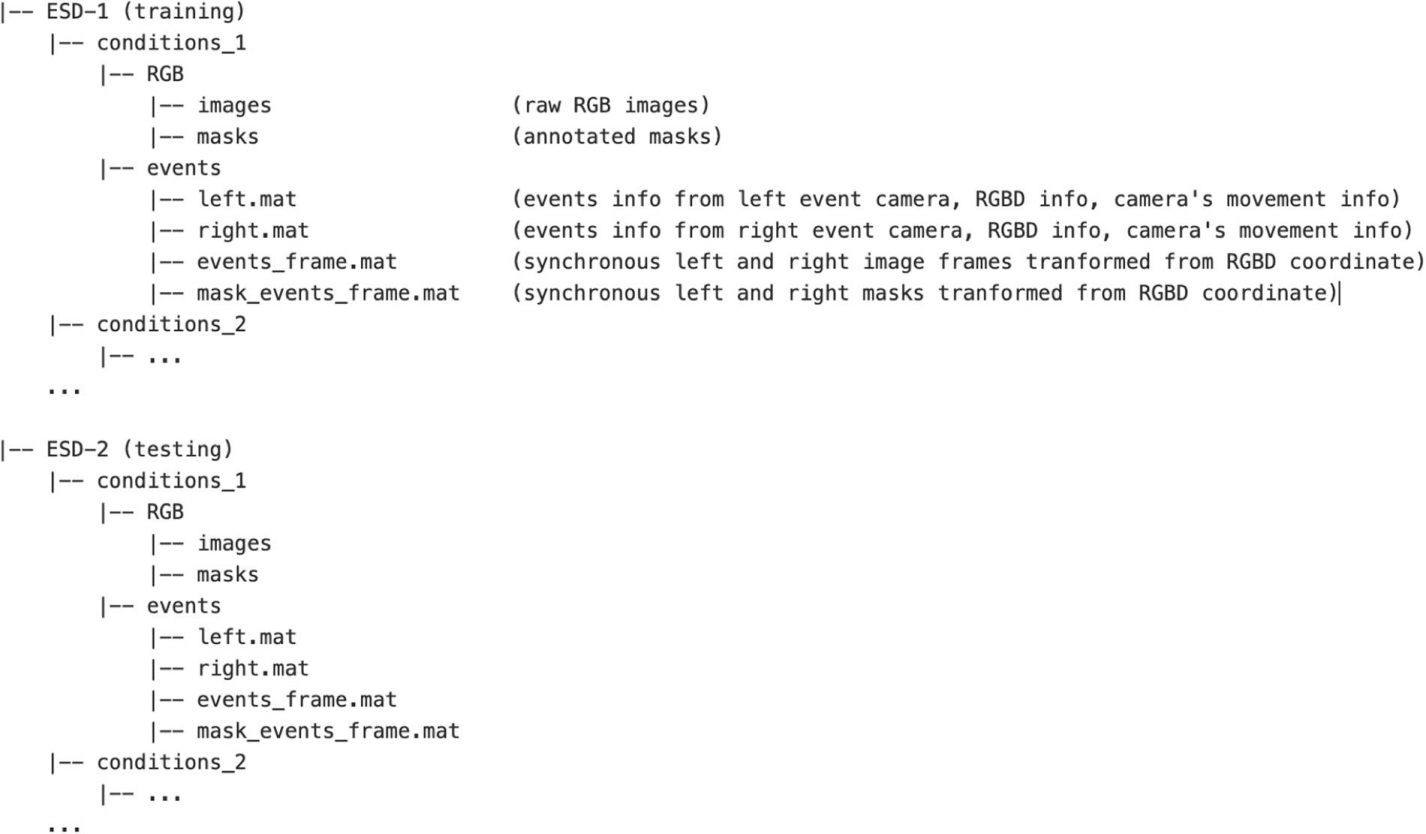
Dataset structure. Each sequence was recorded in the “events” subfolder under different experimental conditions with a unique name under the training or testing path. Event-related and frame-related information is stored under “events” and “RGB” folders, respectively. Particularly, raw images and annotated masks are contained in the “RGB” subfolder under different experimental conditions. Events with RGBD information of both event cameras, image, and mask frames converted from RGBD coordinates and cameras’ movement are recorded under the “events” folder.

All of the ESD^47^ is available at Figshare, whose structure is demonstrated in Fig. 8.

### Data format

In each sequence, event-related data is stored in four distinct files within the “events” folder, which correspond to specific conditions. The “left.mat” file contains events information from left *Davis 346c*, RGBD information from *Intel 435*, and data regarding the movement of the cameras. Similarly, *right.mat* contains events and frames information from the right event-based camera and RGBD camera and information of cameras’ movement. Additionally, synchronous image frames and mask frames converted from RGBD camera coordinates for both event-based camera coordinates are reserved in *events_frame.mat* and *mask_events_frame.mat*. Moreover, the raw RGB images and ground truth masks are also provided in the “RGB” folder of all experimental conditions.

### Dataset challenging factors and attributes

We constructed ESD^47^ dataset with various scenarios and challenges in the indoor cluttered environment. We briefly define the attributes as below, and the symbol * is varying in specific conditions:

- Various number of objects (O*): The complexity of the scene can be affected by the number of objects. Thus, we selected different numbers of objects with various shapes and layouts to increase the diversity of the scenes. Particularly, scenes of 2, 4, 6, 8, and 10 objects were collected in ESD-1. Scenes of 2 and 5 objects were collected in ESD-2.
- Cameras’ moving speed (S*): Motion blur is an open challenge in computer vision tasks. We collected data with different moving speeds (S015: 0.15 *m*/*s*, S03: 0.3 *m*/*s*, S1: 1 *m*/*s*) of cameras to introduce various degrees of motion blur of RGB frames.
- Cameras’ moving trajectory (M*): From the observation, events may not be captured if they are on the edge which is parallel to the camera’s moving direction that is challenging in event-based processing. We introduce this attribute as linear (ML), rotary (MR), and general (linear + rotary (MLR)) moving trajectory to cover all the edge directions.
- Illumination Variant (*L): Illumination has a substantial impact on an object’s appearance and is still an open challenging problem in segmentation. In the dataset recording, we collected data in low lighting (LL) and normal lighting (NL) conditions.
- Height between tabletop and cameras (*H): It affects the size of the overlap area of stereo cameras. Thus, we introduce it as one of the attributes. The dataset is collected with a higher height (HH) and a lower height (LH) indicating that the sensing areas from the stereo camera are fully overlapped and partially overlapped, respectively.
- Occlusion condition (*O): Occlusion is a classical and challenging scenario in segmentation, that is caused by the integration of objects in the scene. We placed objects with occlusion (OY) and without occlusion (ON).

**Fig. 9:**
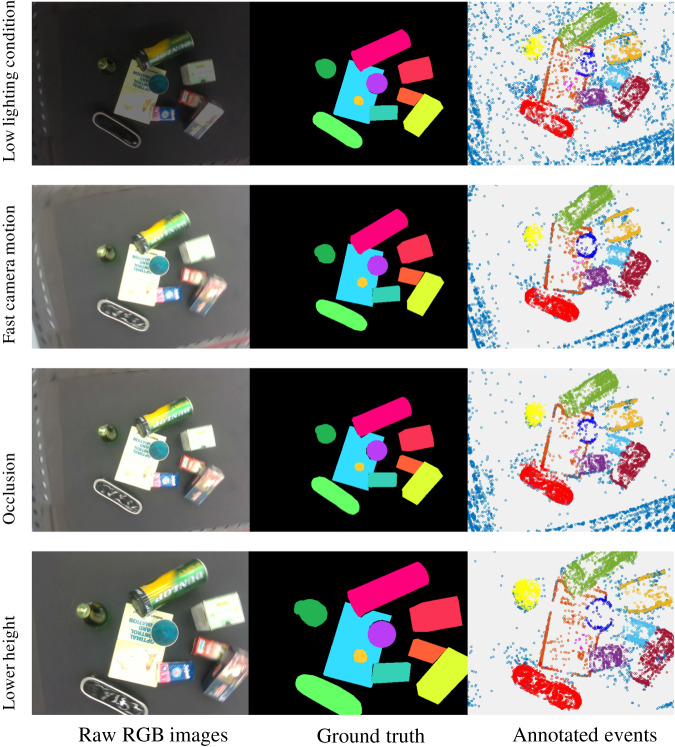
Sample images of RGB, masks and annotated events are selected from our proposed ESD^47^ dataset. (**a**) Shows tabletop objects under low lighting conditions. (**b**) Shows the motion blur scenarios because of the fast camera motion with 1 m/s speed. (**c**) Shows the objects are occluded by others. (**d**) Shows the lower height of cameras with 0.62 m from the tabletop. Different colors in the RGB ground truth and annotated event masks mean different labels. Better view in color.

**Fig. 10:**
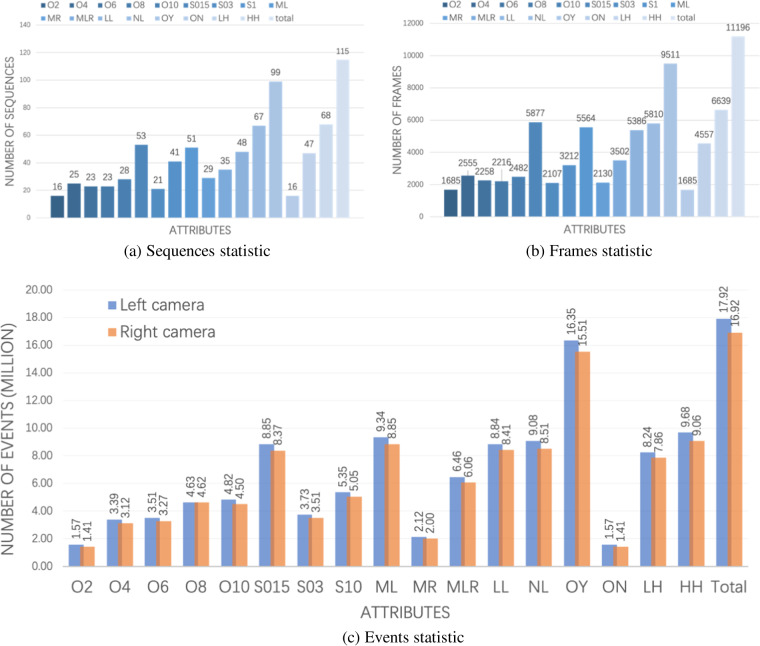
ESD-1 statistic: sequences (**a**), frames (**b**) and events (**c**) statistic in terms of attributes. ML, MR and MLR indicate linear, rotation and hybrid moving types; LN and LL represent normal and low light conditions; S015, S03, and S1 describe the camera’s moving speed of 0.15 m/s, 0.3 m/s and 1 m/s; Similarly, O2, O4, O6, O8 and O10 express sequences of 2–10 objects; The occlusion cases are with and without occlusion referred as OY and ON, respectively. Additionally, the total quantities of sequences, frames and events are also presented in (**a**–**c**), respectively. Better viewed in color.

**Fig. 11:**
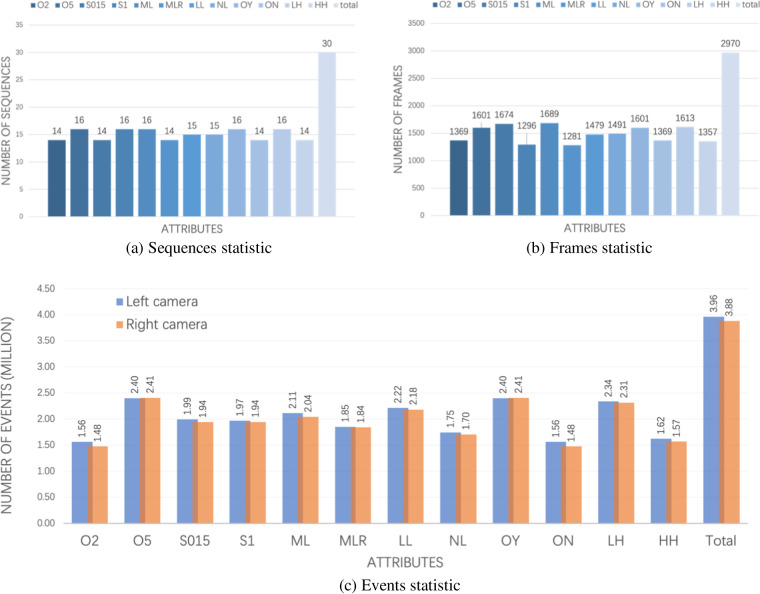
ESD-2 statistic: sequences (**a**), frames (**b**) and events (**c**) statistic in terms of attributes. ML and MLR indicate linear and hybrid moving types; LN and LL represent normal and low light conditions; S015 and S1 describe the camera’s moving speed of 0.15 m/s and 1 m/s; Similarly, O2 and O5 express sequences of 2 and 5 objects; The occlusion cases are with and without occlusion referred as OY and ON, respectively. Additionally, the total quantities of sequences, frames and events are also presented in (**a**–**c**), respectively. Better view in color.

Sample images taken from our proposed ESD^47^ dataset with various attributes are shown in Fig. 9. To address the needs of applications involving unknown objects, two distinct subsets were collected, namely ESD-1 and ESD-2, each featuring different sets of objects. ESD-2 serves as a dataset for testing the performance of models with unknown objects. A total of 115 sequences are collected and labeled in ESD-1, and their attributes are statisticized in Fig. 10 including different light conditions, moving speed, moving trajectories and objects with occlusion. ESD-2 comprises 30 sequences with corresponding data statistics illustrated in Fig. 11. Each sequence in both sub-datasets encompasses eight key aspects: end effector’s pose and moving velocity, RGB frames and depth maps from D435, RGB frames and events stream from left and right Davis 346 C.

## Technical Validation

### Evaluation metrics

Our dataset ESD^47^ provides labels of events for individual objects, making it suitable for instance segmentation tasks. Additionally, ESD^47^ includes objects from various categories, rendering it useful for semantic segmentation as well.In this work, we assess our dataset by applying instance and semantic segmentation methods. Standard segmentation metrics, specifically accuracy and mean Intersection over Union (mIoU), are employed to quantify the testing results. Pixel accuracy, as defined in Eq. 6, measures the percentage of pixels correctly classified.

6 $$Acc(p,p{\prime} )=\frac{1}{N}\mathop{\sum }\limits_{i}^{N}\delta ({p}_{i},{p}_{i}^{{\prime} })$$

where *p*, *p*′, *N*, and *δ* represent the ground truth image, the predicted image, the total number of pixels, and Kronecker delta function, respectively. However, its descriptive power is limited for cases with a significant imbalance between foreground and background pixels. Therefore, mIoU is also utilized in this work as the evaluation metric due to its effectiveness in dealing with imbalanced binary and multi-class segmentation. Mean IoU (mIoU) is calculated across classes as Eq. 7:

7 $$mIoU(p,p{\prime} )=\frac{1}{C}\mathop{\sum }\limits_{i}^{C}\frac{\mathop{\sum }\limits_{i}^{N}\delta ({p}_{i,c},1)\delta ({p}_{i,c},{p}_{i,c}^{{\prime} })}{max(1,\delta ({p}_{i,c},1)+\delta ({p}_{i,c}^{{\prime} },1))}$$

where *C* denotes the number of classes. If a pixel *i* of prediction or ground truth belongs to a certain class c, *p*_*i, c*_ and *p*′_*i, c*_ are 1; otherwise, *p*_*i, c*_ and *p*′_*i, c*_ are 0.

### Segmentation on RGB images

The approaches for RGBD instance segmentation are sophisticated, so we selected several well-known and widely used methods to evaluate our manually labeled RGB frames, such as FCN^19^, U-NET^20^, and DeepLab^21^. The testing results of ESD-1 and ESD-2 datasets using mIoU metrics is 59.36% on FCN, 64.19% for U-Net, and 68.77% for DeepLabV3+. Moreover, the segmentation results on other public conventional datasets MSCOCO^17^, PascalVoc^13^, and CityScape^18^ are also listed in Table 1. When comparing the segmentation results on known objects from ESD-1 to those of most publicly available datasets, both accuracy and mIoU scores appear lower. This discrepancy can be attributed to the shuffling of RGB frames within the sequences, which leads to image blurring when the camera is in motion. However, it is worth noting that, in contrast to other datasets with complex backgrounds, ESD, which is designed specifically for tabletop objects, offers a setting that is relatively more conducive to distinguishing between foreground and background. For this reason, the segmentation results on RGB images from MSCOCO dataset are comparatively lower. On the other hand, these evaluation results underscore the challenges posed by the RGB component of our dataset. These challenges arise not only from object occlusions but also from the impact of motion blur. In addition, the performance of all testing results on unknown objects from the ESD-2 sub-dataset exhibits a reduction of approximately 30%.

**Table 1:** Evaluation results of the state-of-the-art segmentation networks FCN, U-Net and DeepLab on RGB frames from ESD^47^.

Datasets	FCN^19^		U-Net^20^		DeepLab^21^	
	Acc	mIoU	Acc	mIoU	Acc	mIoU
ESD-1 Known obj (ours)	81.37	59.36	86.27	64.19	90.59	68.77
ESD-2 Unknown obj (ours)	64.21	32.79	69.05	40.70	72.16	43.04
MSCOCO^17^	71.6	31.43	77.2	47.21	79.13	58.01
PascalVoc^13^	87.09	62.20	92.05	72.70	96.52	87.30
Cityscape^18^	84.3	65.30	89.07	73.50	93.17	82.10

Furthermore, benchmarks of the same networks on other public datasets MSCOCO, PascalVoc and CityScape are also provided.

### Segmentation on events data

As mentioned in *Background & Summary* section, there are several approaches for semantic segmentation of autonomous driving, such as EV-SegNet (2019)^24^, VID2E (2019)^25^, EVDistill (2021)^26^, EV transfer (2022)^27^, and ESS (2022)^28^. Given the limited availability of deep learning-based approaches for instance segmentation utilizing neuromorphic vision, transfer learning from semantic segmentation models presents a viable strategy for tackling instance segmentation tasks. However, some of these models are not fully open-sourced, and pre-trained models are not readily provided, which can hinder their implementation and testing on our datasets. Therefore, we applied transfer learning on EV-SegNet and ESS by unfreezing the last 4 convolution layers of the encoder, the entire decoder module, and the classifier. As a result, we achieved testing accuracy rates of 76.98% and 81.59% for EV-SegNet and ESS, respectively. Nevertheless, the mIoU scores for EV-SegNet and ESS stood at 7.73% and 8.92%, as detailed in the Table 2.

**Table 2:** Quantitative evaluation results of transfer learning of EV-SegNet^24^ and ESS^28^ on our proposed dataset ESD^47^ using accuracy and mIoU.

Terms	EV-SegNet^24^		ESS^28^	
	Acc	mIoU	Acc	mIoU
ESD^47^	76.98	7.73	81.59	8.92

Compared to the mIoU results on the autonomous driving dataet DDD17^31^ which is 51.76% and 51.57%, the aforementioned results demonstrate unsatisfactory segmentation performance on our dataset. This discrepancy may be attributed to the substantial differences in features between our tabletop objects dataset and the autonomous driving dataset. Our ESD^47^ captures events of static objects and backgrounds using moving cameras, which provides homogeneous features on events. However, the DDD17 dataset records dynamic moving objects such as on-road vehicles and pedestrians, providing additional features including various moving velocities, directions, and postures. Moreover, this comparison underscores the inherent challenges posed by our dataset, given the homogeneity of features among tabletop objects and the background, which contributes to the complexity of the segmentation task.

### Segmentation on integrated RGB and events data

Since the transfer learning of the event-based semantic segmentation approach fails to provide satisfactory results, we tested our dataset using vision-transformer-based cross-modal fusion networks SA-GATE^48^ and CMX^29^ to extract features from RGB frames and events stream. The quantitative testing results of both known and unknown objects are listed in Table 3. In the case of known object segmentation, both models demonstrate the capability to achieve high accuracy and mIoU scores in their predictions. When compared to the testing results based solely on pure RGB frames, segmentation using the integrated RGB and events data achieved even more accurate results. This enhancement can be attributed to the complementary features that are extracted and fused from both RGB frames and events stream. However, the performance of segmenting unseen objects drops dramatically by 80.66% and 80.00% using SA-GATE and CMX, respectively, indicating the challenge of unknown object segmentation. This sharp decline highlights the challenge associated with segmenting unknown objects.

**Table 3:** Quantitative evaluation results of SA-GATE^24^ and CMX^29^ on RGB frames and events stream from our proposed dataset ESD^47^ using accuracy and mIoU.

Datasets	SA-GATE^48^		CMX^29^	
	Acc	mIoU	Acc	mIoU
ESD-1 (Known objects)	91.53	84.08	94.58	85.81
ESD-2 (Unknown objects)	73.04	16.26	76.78	18.90

**Fig. 12:**
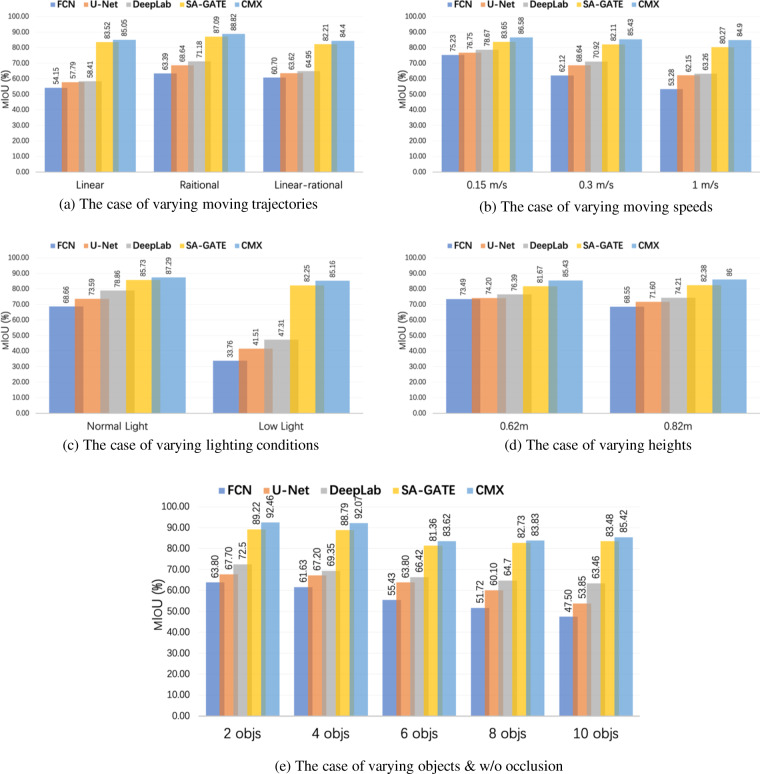
The testing results in terms of different attributes: (**a**) moving trajectories of cameras, (**b**) moving speed of cameras, (**c**) lighting condition, (**d**) the distance between table and cameras, and (**e**) clutter objects w/o occlusion. Better view in color.

Besides, the testing results are also shown in Fig. 12 for comparison.

#### Varying moving trajectories

We also conducted experiments to compare the performance of the methods according to the type of robotic arm movement or direction of the camera motion. There are three types of robotic arm movement, i.e. linear, rotational, and linear-rotational. In the case of an event-based vision sensor, the direction of motion is an important factor as object edges perpendicular to the motion direction are relatively more exploited than the parallel edges. The impact of the phenomenon evident in Fig. 12a in terms of the accuracy of segmentation. In general, rotational motion provides richer information as compared to linear motion. Consequently, the fusion of event frames with RGB, as exemplified by the CMX model, delivers the highest accuracy, at 88.82%. This accuracy level surpasses the results of linear and partial linear motion by 3.77% and 4.42%, respectively.

#### Moving speed of cameras

The testing results under various camera movement speeds are illustrated in Fig. 12b. In comparison to approaches solely utilizing RGB frames, as discussed in Section [insert section number], CMX, when applied to both RGB images and events data, exhibits the highest mIoU, reaching 85.58% for a camera speed of 0.15 m/s. However, this score diminishes slightly to 84.90% when the camera speed is increased to 1 m/s. This demonstrates the significant impact of event-based vision at high speeds, which aids in recovering information along contours and mitigates the adverse effects of motion blur in RGB frames.

#### Varying lighting conditions

Figure 12c demonstrates the testing results under conditions of varying lighting conditions. The integration of event data substantially improves the performance of the hybrid model, which employs both RGB frames and event information, outperforming other approaches relying solely on RGB images. This improvement is particularly notable in low-light conditions, where the mIoU scores for traditional RGB testing often fall below 50%. The diminished performance in conventional RGB images is primarily due to the reduced perception quality under low-light conditions. However, when events data is integrated, the segmentation mIoU surges to approximately 85%. This notable enhancement can be attributed to the event camera’s high sensitivity to changes in light intensity, enabling more robust performance even in challenging lighting scenarios.

#### Varying distance between cameras and table

The distance between the camera and the object is varied between 62 *cm* and 82 *cm*, the results are illustrated in Fig. 12d. Although, there is a minimal impact of the camera and object distance on the accuracy of all the models, yet the effect in the performance of the CMX is 0.67% compared to the DeepLabV3 2.18%.

#### Varying objects/occlusion

The segmentation results for different numbers of objects are shown in Fig. 12e. The scenario of two objects also indicates the condition without occlusion, and scenarios of more than 2 objects represent the occluded condition as depicted in Fig. 6. As the number of objects in the scenarios increases, the complexity of the task also grows. Thus, we can observe a decrease in the mIoU score for all models when dealing with RGB frames as the number of objects increases. However, the testing results of both cross-modal networks exhibit a trend resembling a U-shape. The lowest mIoU value is observed in the 6-object scenario. This phenomenon may be attributed to the specific configuration where one object is fully stacked on top of another object, leading to a particularly challenging segmentation scenario.

## Usage Notes

This dataset serves as a data source for the exploration of event-based tabletop object segmentation within the context of robotic grasping tasks. To the best of our knowledge, it represents the inaugural dataset to provide event-wise depth information, segmentation labels, and corresponding RGBD frames. Nevertheless, it is imperative to acknowledge that our dataset does possess certain limitations, such as the simple background and objects. Therefore, this dataset ESD constructed can be continually expanded to cover more subjects and more challenging scenes for the development of segmentation algorithms as well as depth estimation tasks. In addition, more ground truth information such as the bounding box and classes can be added to facilitate the use of the dataset for various event-based applications such as object detection, semantic segmentation, and object tracking.

Furthermore, based on the validation results of state-of-the-art algorithms applied to our dataset, it is evident that further explorations in algorithm development are essential to attain successful event-based object segmentation, particularly when relying on pure event data. Therefore, in the future, we intend to delve into the development of learning methods, such as graph neural networks and spiking neural networks, for the event-based segmentation of tabletop objects, with a focus on facilitating robotic grasping tasks.

## Data Availability

All the events were automatically labeled by the Matlab programs. All Matlab codes are available on GitHub^49^ https://github.com/yellow07200/ESD_labeling_tool.
